# Supplementary material for: Identification of nine new susceptibility loci for endometrial cancer
Source: Nat Commun. 2018 Aug 9;9:3166. doi: 10.1038/s41467-018-05427-7 (PMC6085317; doi:10.1038/s41467-018-05427-7)
Supplement: Supplementary file 1 — Supplementary Information [file 41467_2018_5427_MOESM1_ESM.pdf]

Supplementary Information

Identification of nine new susceptibility loci for endometrial cancer. O'Mara et al.

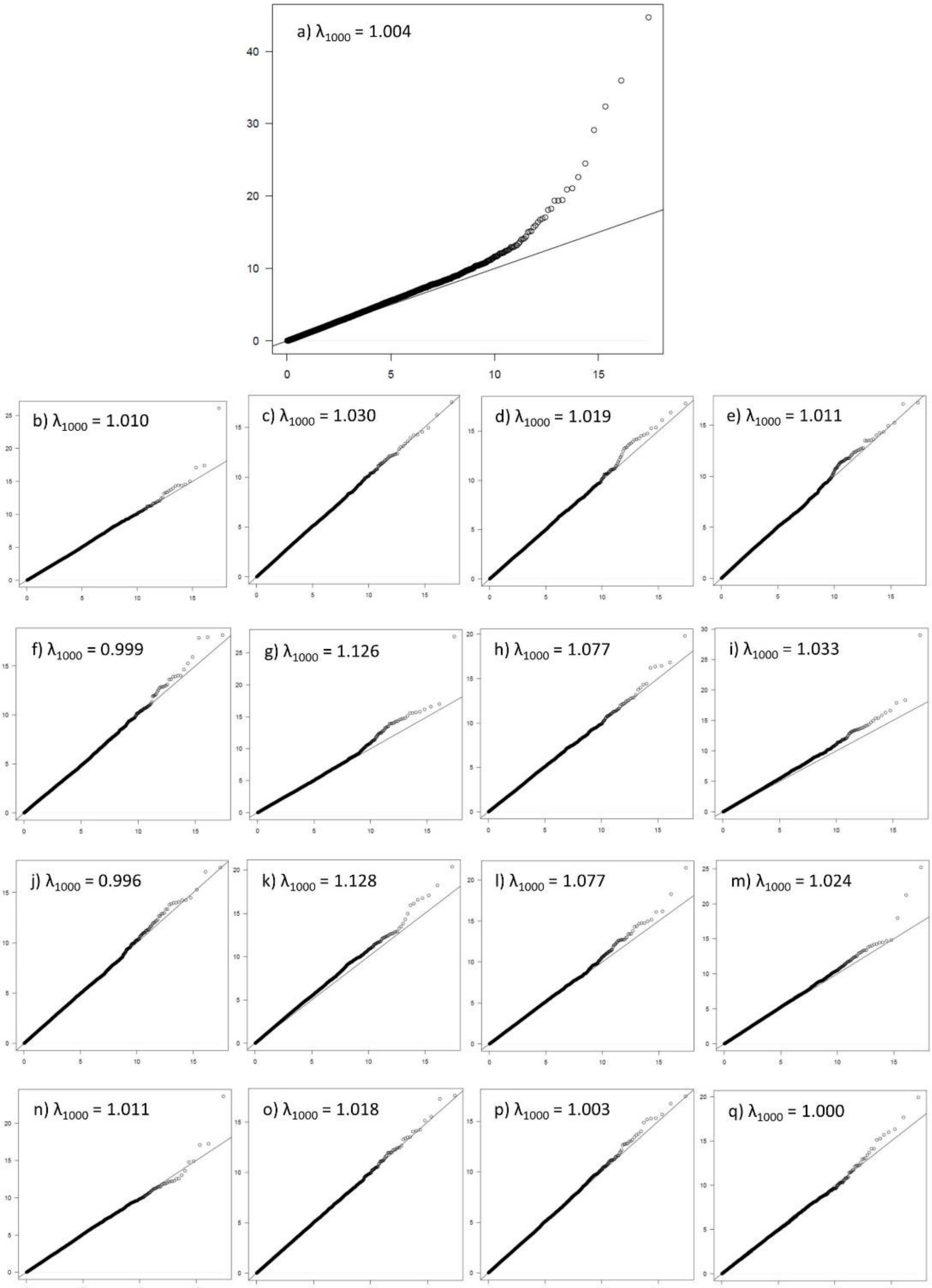

Supplementary Fig. 1. Quantile Quantile plots (33,278 uncorrelated SNPs). a) Meta-analysis of all studies b) NSECG GWAS c) SEARCH GWAS d) ANECS GWAS e) E2C2 OmniExpress f) E2C2 Illumina 660W g) iCOGS Germany h) iCOGS Norway i) iCOGS UK j) OncoArray Australia k) OncoArray Belgium l) OncoArray Germany m) OncoArray Sweden n) OncoArray UK o) OncoArray USA p) WHI q) UK

Biobank.

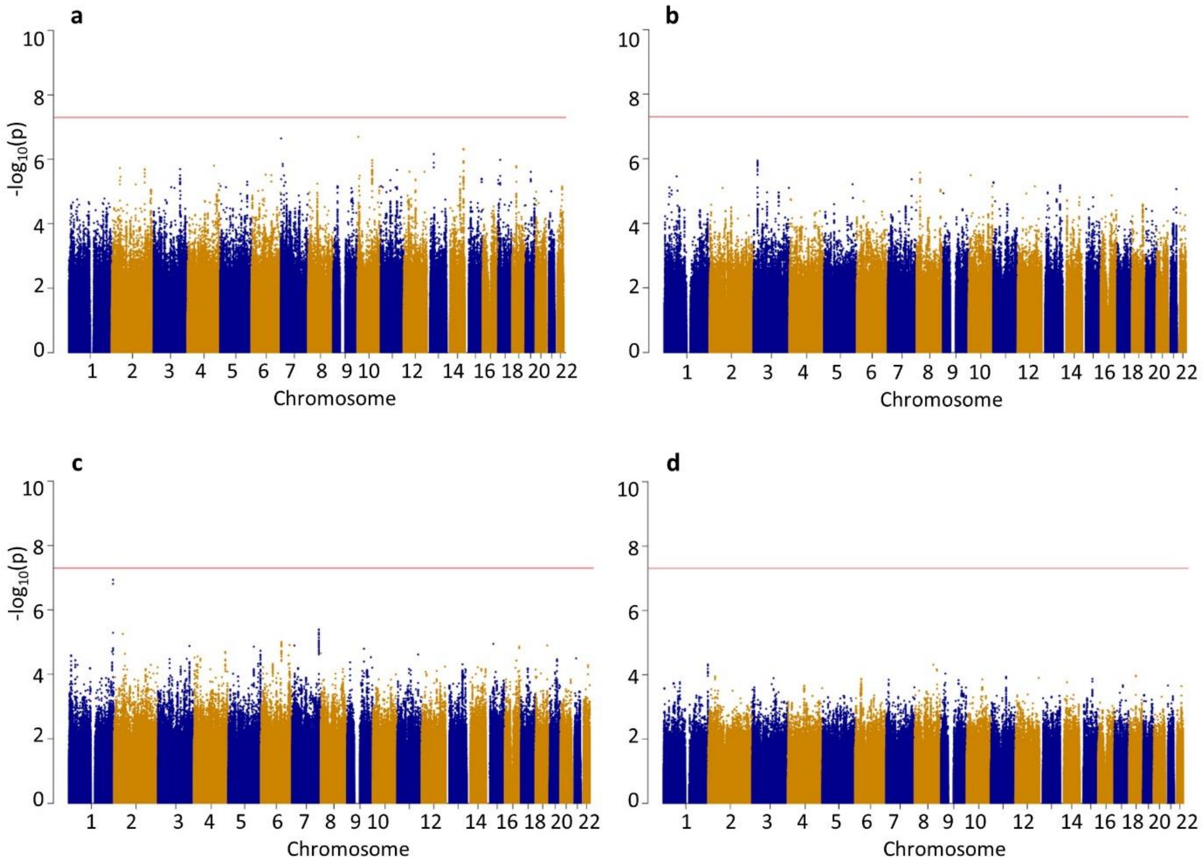

Supplementary Fig. 2. Manhattan plot of the analyses of specific endometrial cancer non-endometrioid histological subtypes comparing to 108,979 controls. Genetic variants are plotted according to chromosome and position (X-axis) and statistical significance (Y-axis). The red line marks the $5x10^{-8}$ GWAS significance threshold. a) Serous carcinoma (434 cases) b) Carcinosarcoma (164 cases) c) Clear cell carcinoma (128 cases) d) Mucinous carcinoma (49 cases).

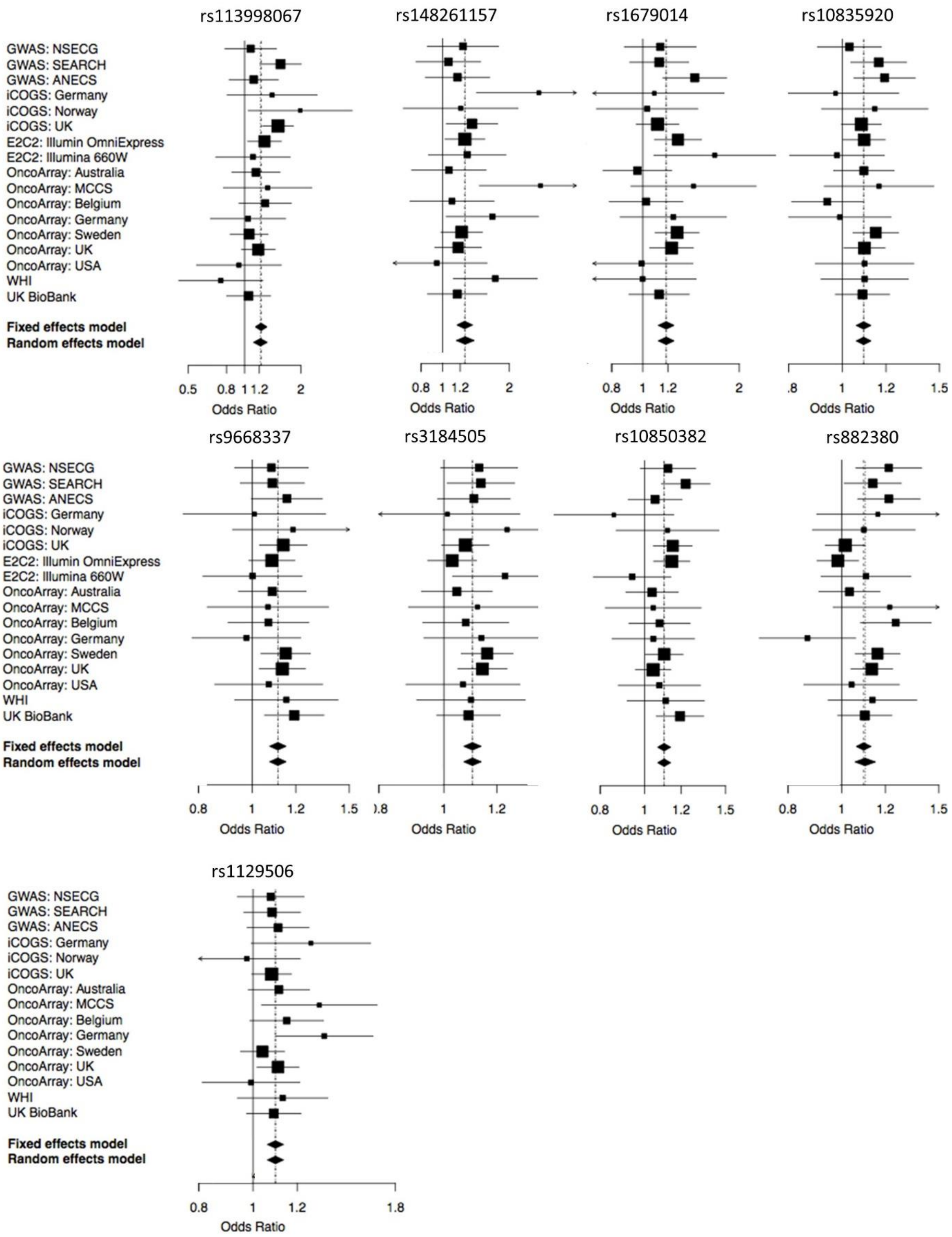

Supplementary Fig. 3. Forest plots for the nine new endometrial cancer susceptibility loci. Details of the individual studies are given in Supplementary Data 1.

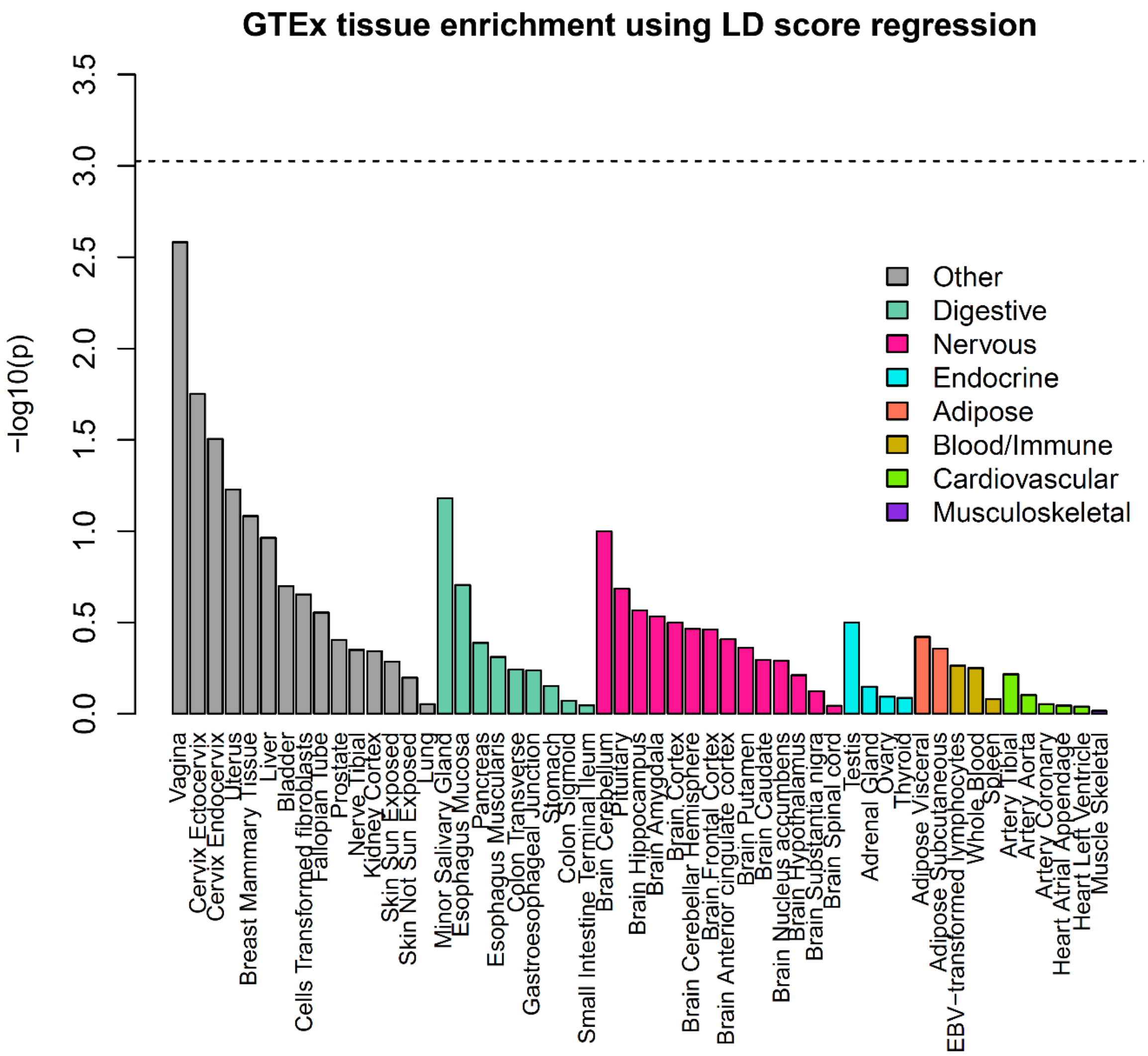

Supplementary Fig. 4. GTEx tissue enrichment using LD score regression. The dashed line represents Bonferroni-corrected significance for the number of tissues tested.

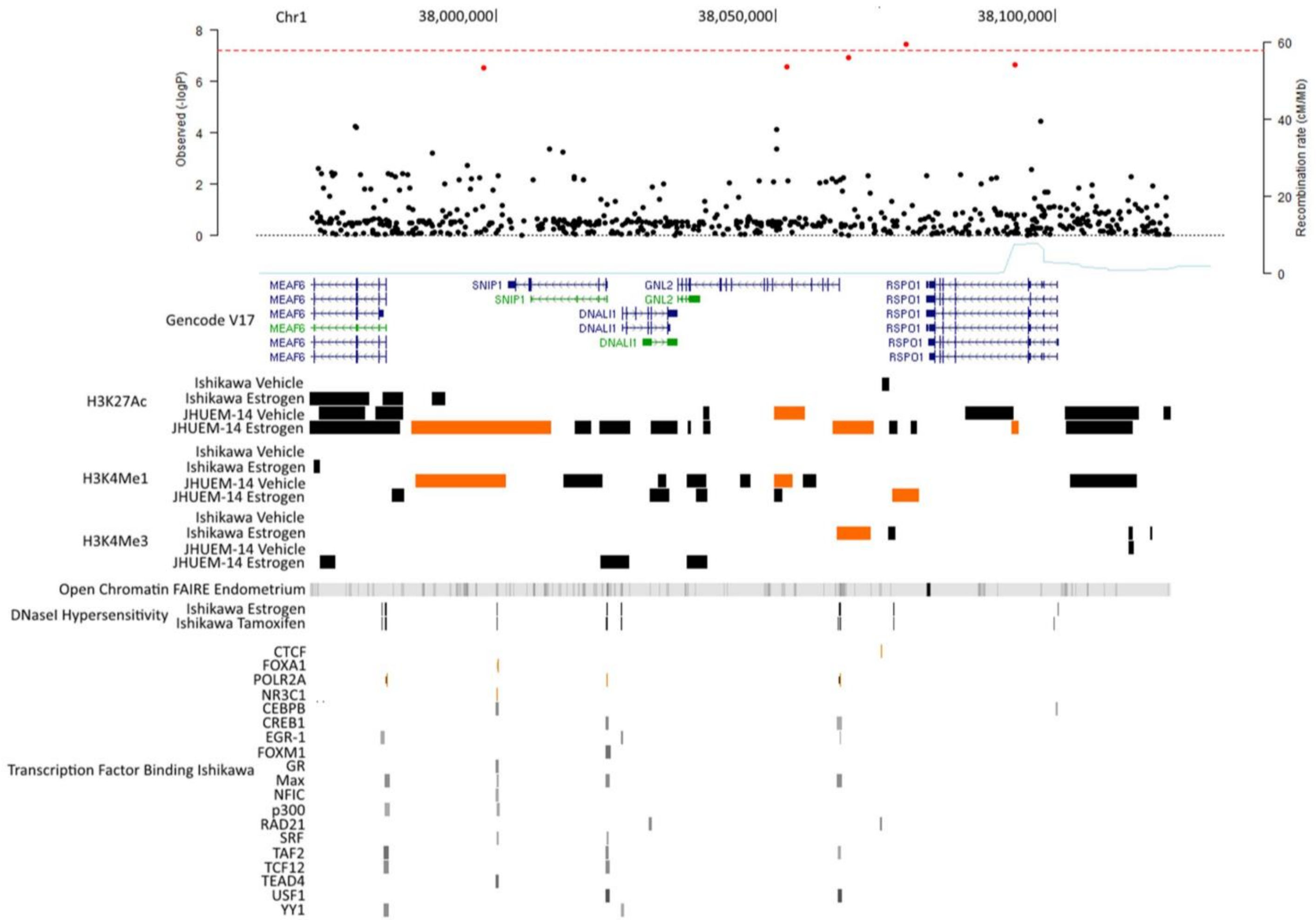

Supplementary Fig. 5. Regional association plots and genomic featured at the 1p34.3 region. ccrSNPs are denoted by red dots. All five ccrSNPs are coincident with histone marks identified in endometrial cancer cells (highlighted in orange). The risk allele of the ccrSNP rs114240971 is associated with increased *CDCA8* expression in all (p=0.0019) and endometrioid (p=0.00078) TCGA endometrial tumours (ST3). rs114240971 is an intergenic variant coincident with histone marks characteristic of enhancers: H3K4Me1 in estrogen-stimulated JHUEM-14 and H3K27Ac in uterus and vagina. *CDCA8* is an essential regulator of mitosis and cell division, and knockdown of expression suppresses cancer cell proliferation [1,2]. In ovarian cancer, and *CDCA8* has been identified as an oncogene [3] and risk variation associates with *CDCA8* expression in TCGA ovarian tumours [4].

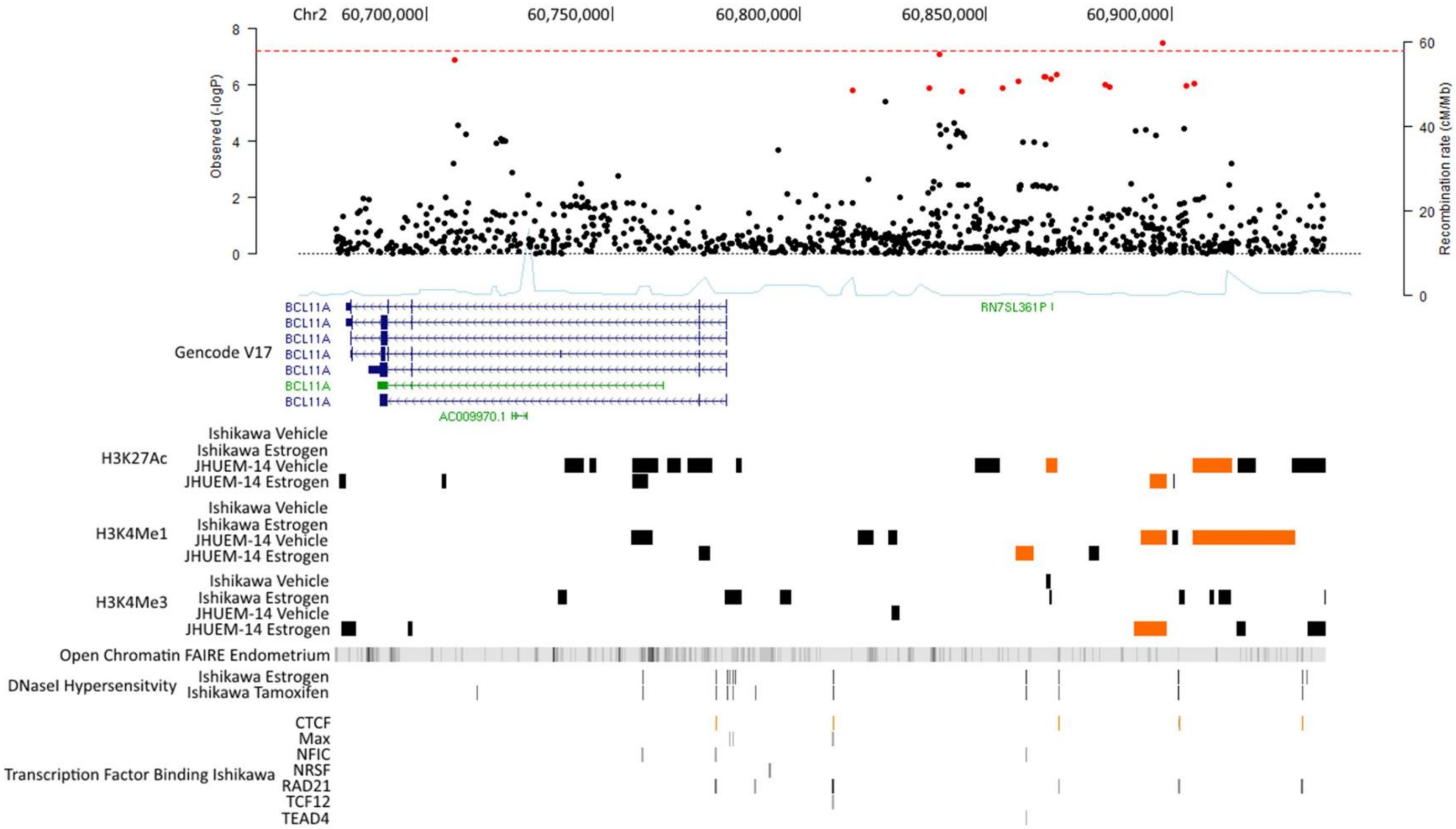

Supplementary Fig. 6. Regional association plots and genomic feature intersects for 2p16.1 region. ccrSNPs are denoted by red dots. Five of the 16 ccrSNPs are coincident with histone marks identified in endometrial cancer cells (highlighted in orange). The lead ccrSNP, rs148261157, is an intergenic variant located in an active chromatin region delineated by H3K27Ac, H3K4Me1 and H3K4Me3 in JHUEM-14 cells.

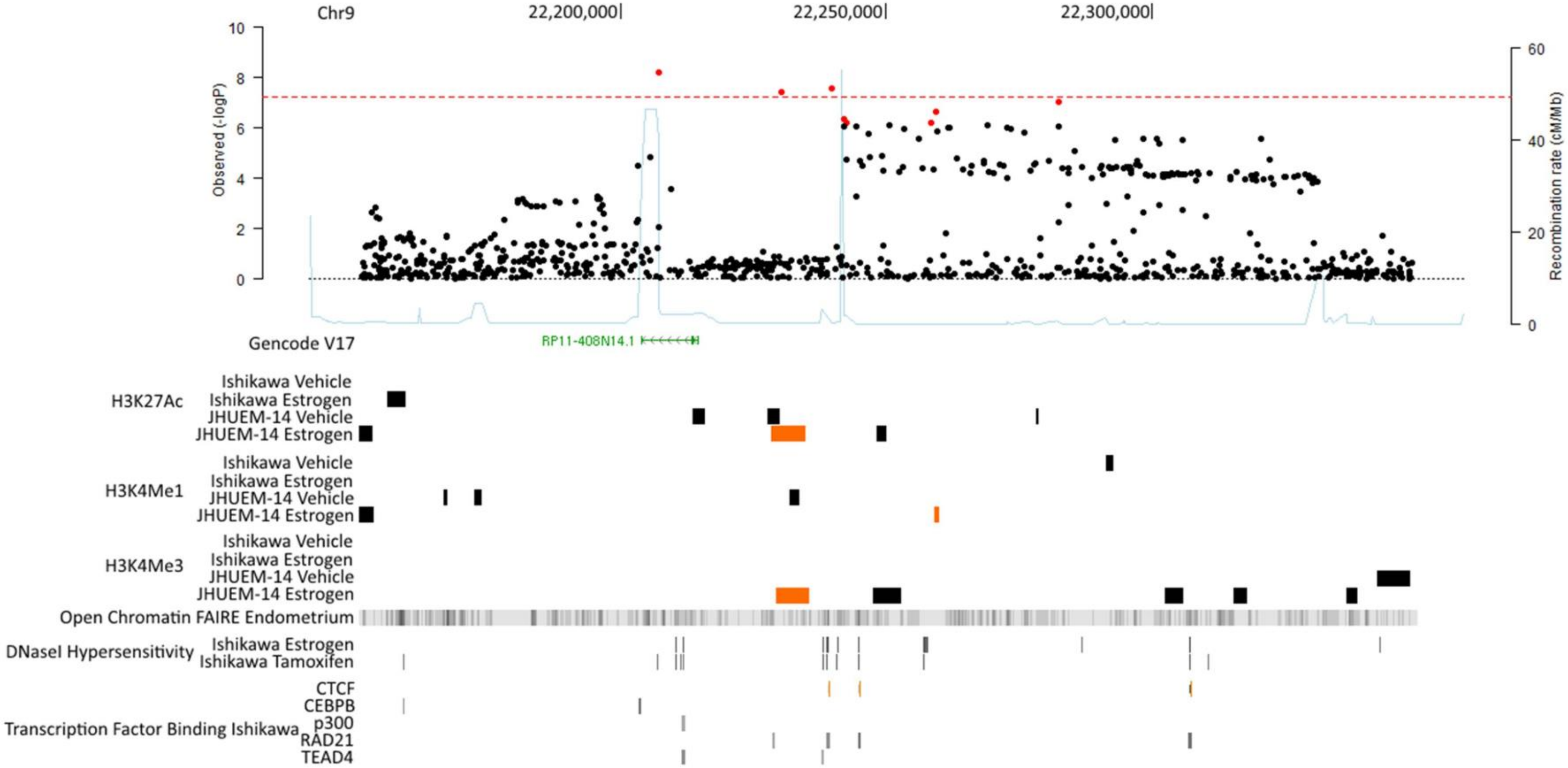

Supplementary Fig. 7. Regional association plots and genomic feature intersects for 9p21.3 region. ccrSNPs are denoted by red dots. Three of the eight ccrSNPs are coincident with DHS or histone marks in endometrial cancer cell lines (histone marks highlighted in orange). The lead ccrSNP, rs1679014, is a lncRNA (RP11-408N14.1) intronic variant located in a DNaseI hypersensitivity site (DHS) in Ishikawa cells.

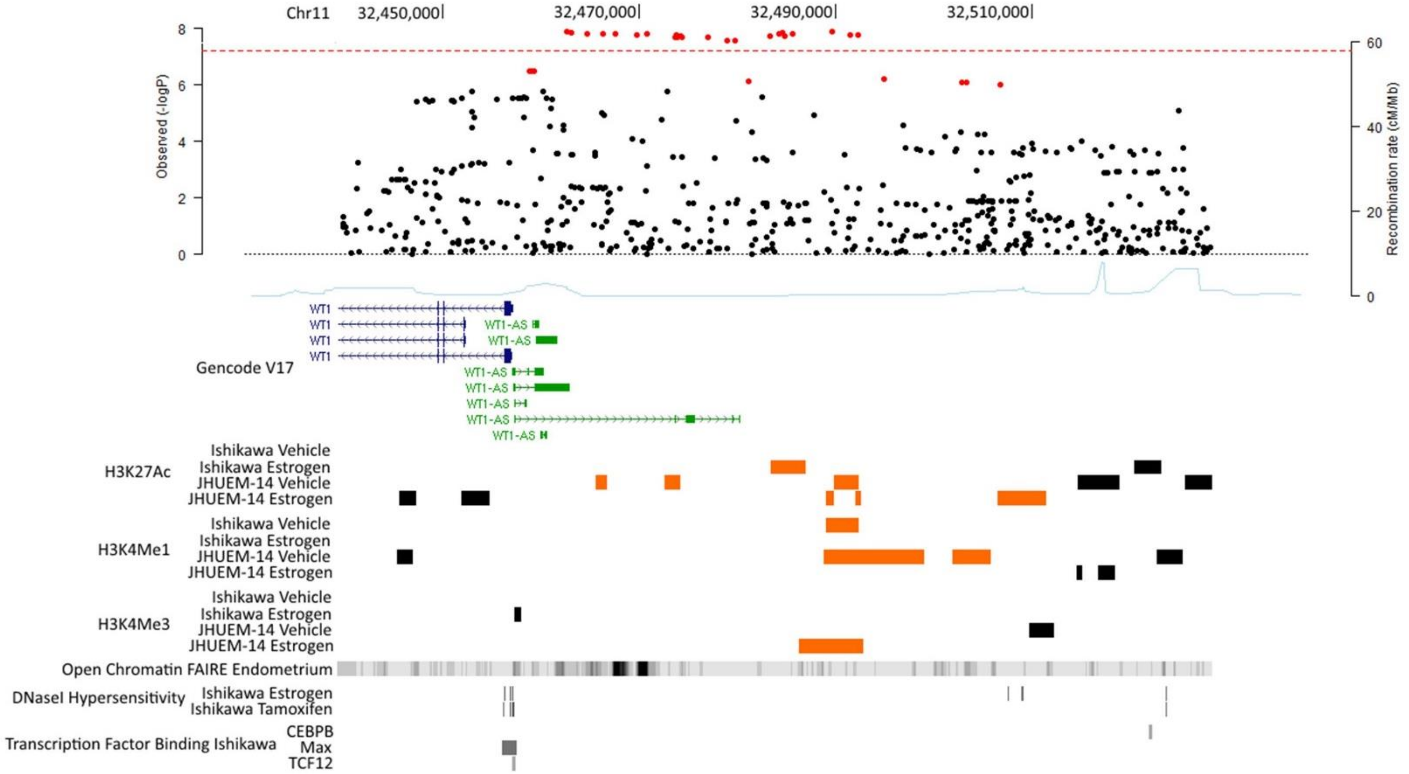

Supplementary Fig. 8. Regional association plots and genomic feature intersects for 11p13 region. ccrSNPs are denoted as red dots. 15 of the 33 ccrSNPs are coincident with histone marks identified in endometrial cancer cell lines (highlighted in orange). Notably, ccrSNPs are associated with the expression of multiple genes in normal and tumour endometrial tissues. The risk alleles of ccrSNPs are associated with increased *CCDC73* in tumour (p=0.039-0.00049) and normal (p=0.048-0.0076) TCGA endometrial tissue (ST3). *CCDC73* encodes coiled-coil domain-containing protein 73 but the function of this protein remains unknown. Risk alleles are also associated with RCN1 expression in normal (p=0.046-0.0052), endometrioid (p=0.034-0.0011) and all tumour (p=0.0056-0.00084) TCGA endometrial tissues (ST3). *RCN1* encodes reticulocalbin 1, a calcium-binding protein that binds oncoproteins such as JAK2 and MYC (https://www.ncbi.nlm.nih.gov/gene/5954). In uterine sarcoma cells, overexpression of RCN1 contributes to doxorubicin-resistance [5] and expression of RCN1 enhances survival of irradiated epithelial cancer cells under hypoxic conditions [6]. The risk alleles of four ccrSNPs are associated with increased *WT1-AS* expression in normal endometrium (p<0.001; ST3). These variants are located ~1kb downstream of a *WT1-AS* promoter delineated by H3K4Me3 in estrogen-treated Ishikawa cells. *WT1-AS* is a long non-coding RNA that overlaps the 5' UTR of *WT1* and strongly correlates with *WT1* expression in TCGA endometrial tumour samples. Indeed, *WT1-AS* can induce *WT1* expression [7] and upregulate WT1 protein levels [8]. *WT1* encodes Wilms tumor protein which appears to act as an oncogene in many tumours and plays an important role in the cellular mesenchymal-epithelial balance [9].

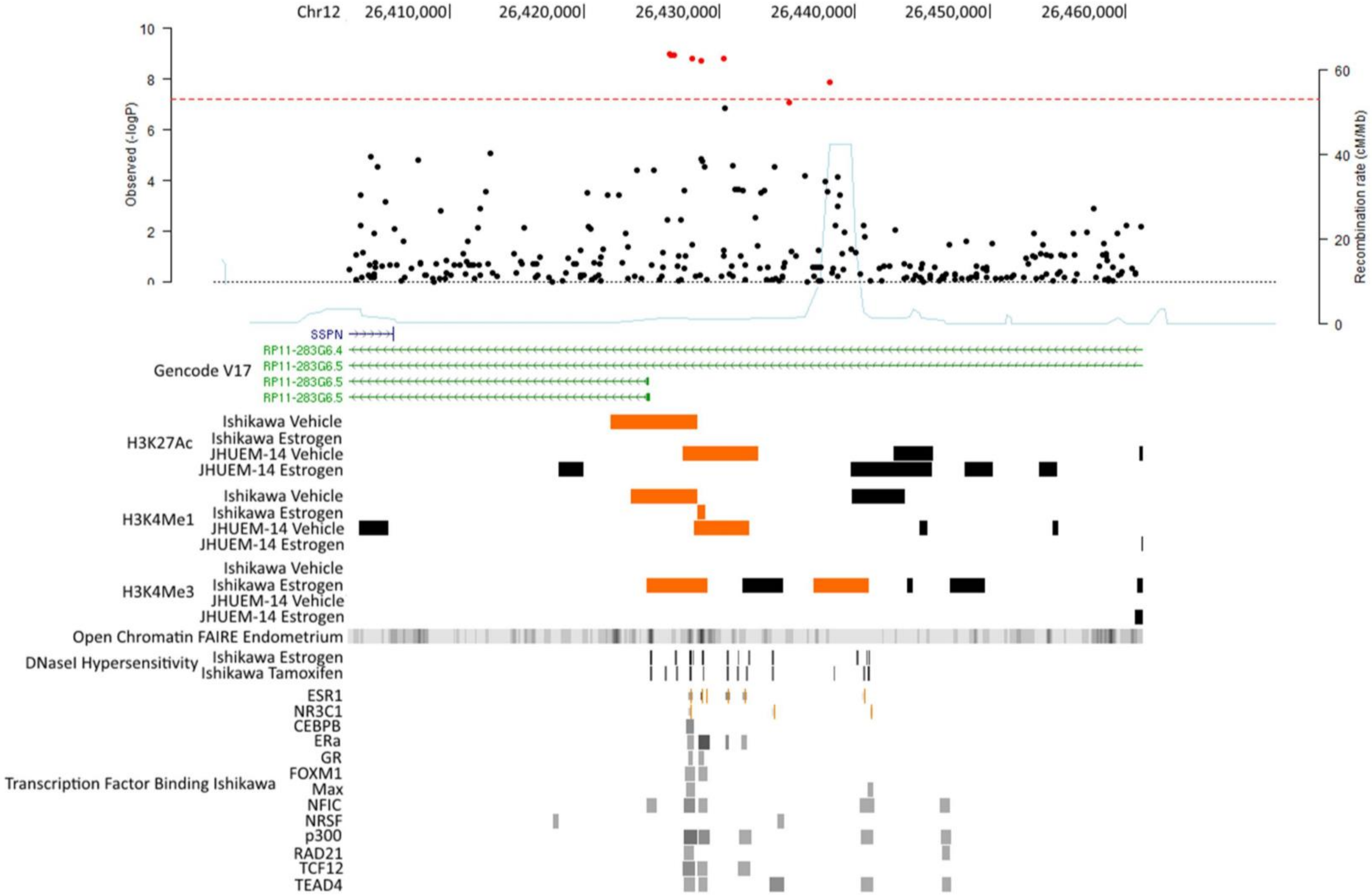

Supplementary Fig. 9. Regional association plots and genomic featured at the 12p12.1 region. All nine ccrSNPs (denoted by red dots) are located in the intronic region of a lncRNA (RP11-283G6.5) that is downregulated in breast tumours[10]. All but one of the ccrSNPs are located in regions of active chromatin delineated by histone marks in endometrial cancer (highlighted in orange). Furthermore, two ccrSNPs, rs7959150 and rs7974900, appear to be located in an endometrial cancer enhancer. These variants are coincident with DHS and/or H3K4Me1 and H3K27Ac enhancer marks in endometrial cancer cells. Moreover, in Ishikawa cells, these variants are coincident with regions bound by transcription factors associated with enhancers such as p300, Era, FOXM1

and RAD21.

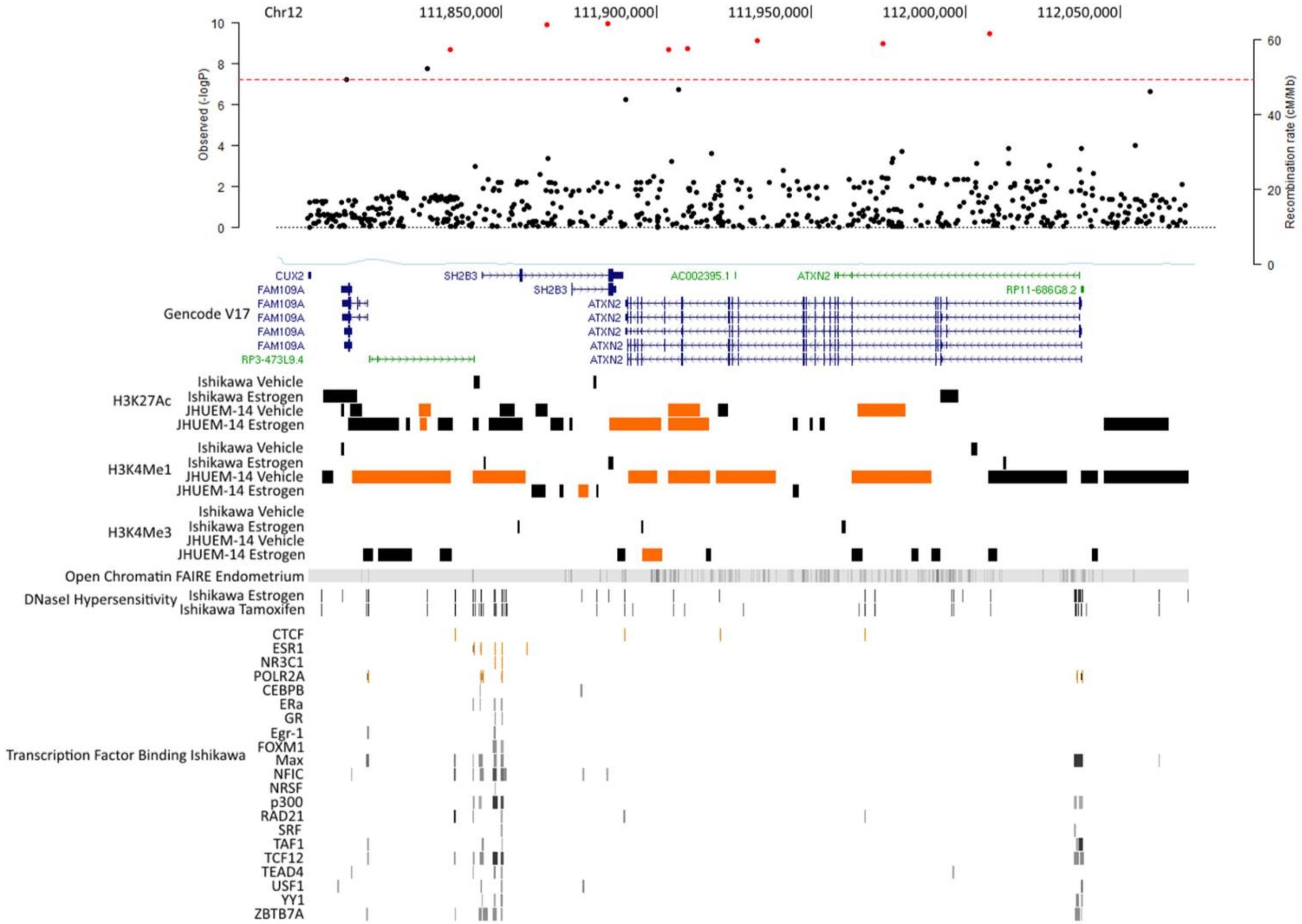

Supplementary Fig. 10. Regional association plots and genomic featured at the 12p24.11 region. ccrSNPs are denoted by red dots. All but one of the eight ccrSNPs are coincident with histone marks identified in endometrial cancer cells (highlighted in orange). The risk alleles of four ccrSNPS are associated with decreased *SH2B3* expression in blood ($p \leq 5.02 \times 10^{-19}$, ST3), including the lead variant at this locus, rs3184504 (Trp262Arg), which causes a substitution in the pleckstrin homology domain of SH2B3. Relevantly, SH2B3 is a negative regulator of the oncoproteins c-KIT and JAK2 and also of processes related to

angiogenesis and neovascularisation [11].

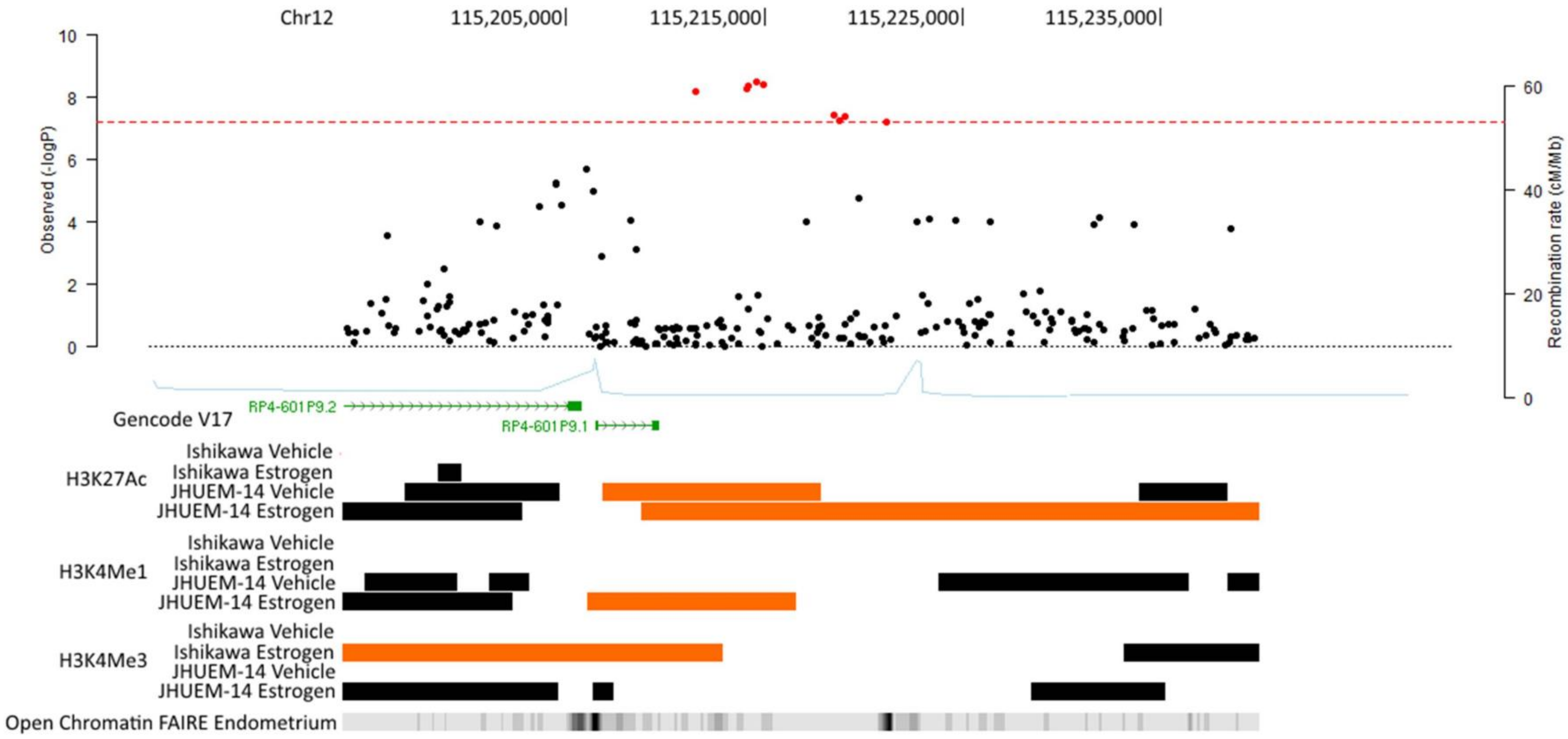

Supplementary Fig. 11. Regional association plots and genomic featured at the 12p24.21 region. All nine ccrSNPs (denoted by red dots) are located in an intergenic region of active chromatin delineated by H3K4Me1, H3K4Me3 and H3K27Ac marks in endometrial cancer cells (highlighted in orange).

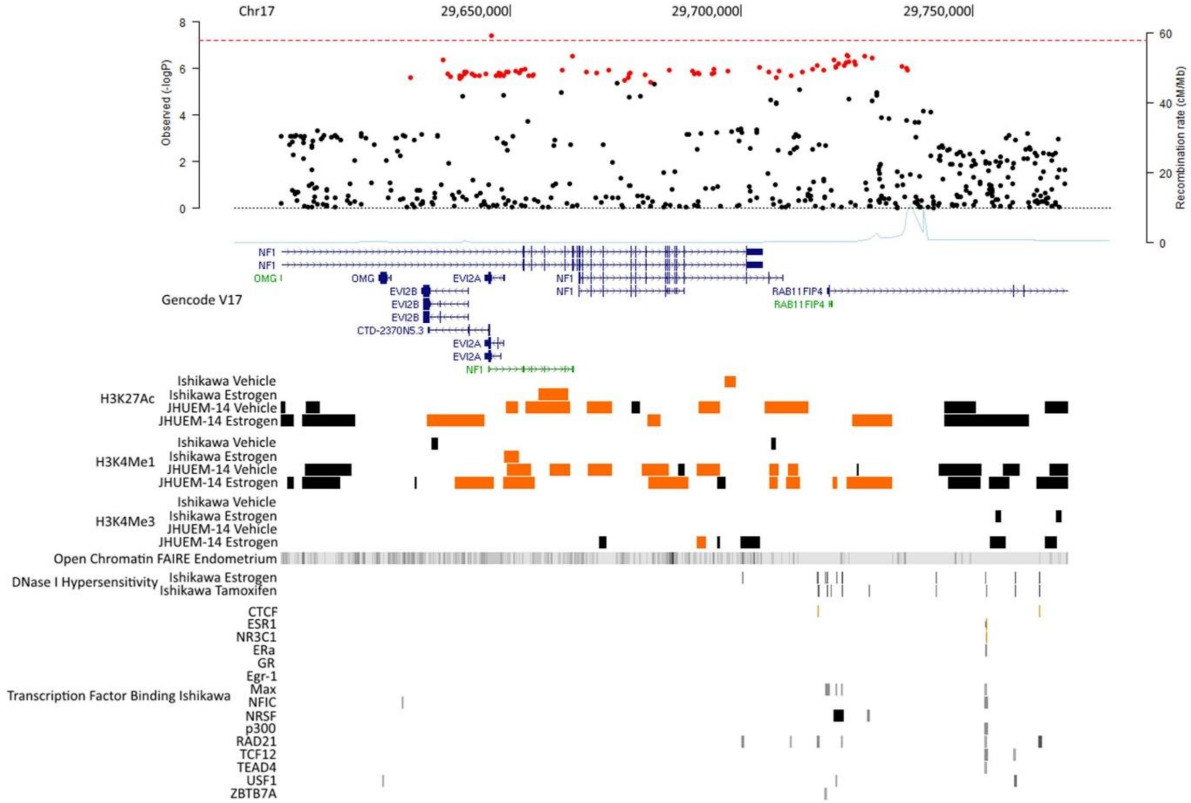

Supplementary Fig. 12. Regional association plots and genomic featured at the 17q11.2 region. ccrSNPs are denoted by red dots. 56 of the 80 ccrSNPs are located in either a DHS or histone mark in endometrial cancer cells (histone marks highlighted in orange). The risk alleles of 40 ccrSNPs are associated with decreased *EVI2A* and *NF1* expression in blood ($p \leq 1.68 \times 10^{-36}$; ST3), while the lead (*EVI2A* Ser23Ser) ccrSNP associated with increased *NF1* expression in TCGA endometrial tumours ($p = 7.28 \times 10^{-6}$; ST3). Little is known about the function of EVI2A although it has been proposed that it may play a role in interactions between cancer cells and the extracellular matrix [12]. *NF1* encodes neurofibromin, a tumour suppressor that acts a negative regulator of the oncogenic RAS proteins [13]. Consistent with the function of NF1, a number of *NF1* truncating (putative driver) mutations have been observed in TCGA endometrial tumours, as well as significant deletion in a region encompassing *NF1* and *EVI2A* (GISTIC q-value = 7.19×10^{-8}; data accessed from cBioPortal). Nominal associations were also observed between the risk alleles of 26 ccrSNPs and decreased *SUZ12* expression in normal endometrium (p=0.0076-0.0026, ST3). *SUZ12* encodes a transcription factor required for transcriptionally repressive H3K27 methyltransferase activity [14] and is located at a recurrent chromosomal breakpoint in endometrial stromal sarcoma where *SUZ12* fuses with *JAZF1* [15]. This *SUZ12-JAZF1* fusion abolishes H3K27 methyltransferase activity and has an oncogenic effect by activating genes that are normally repressed [16].

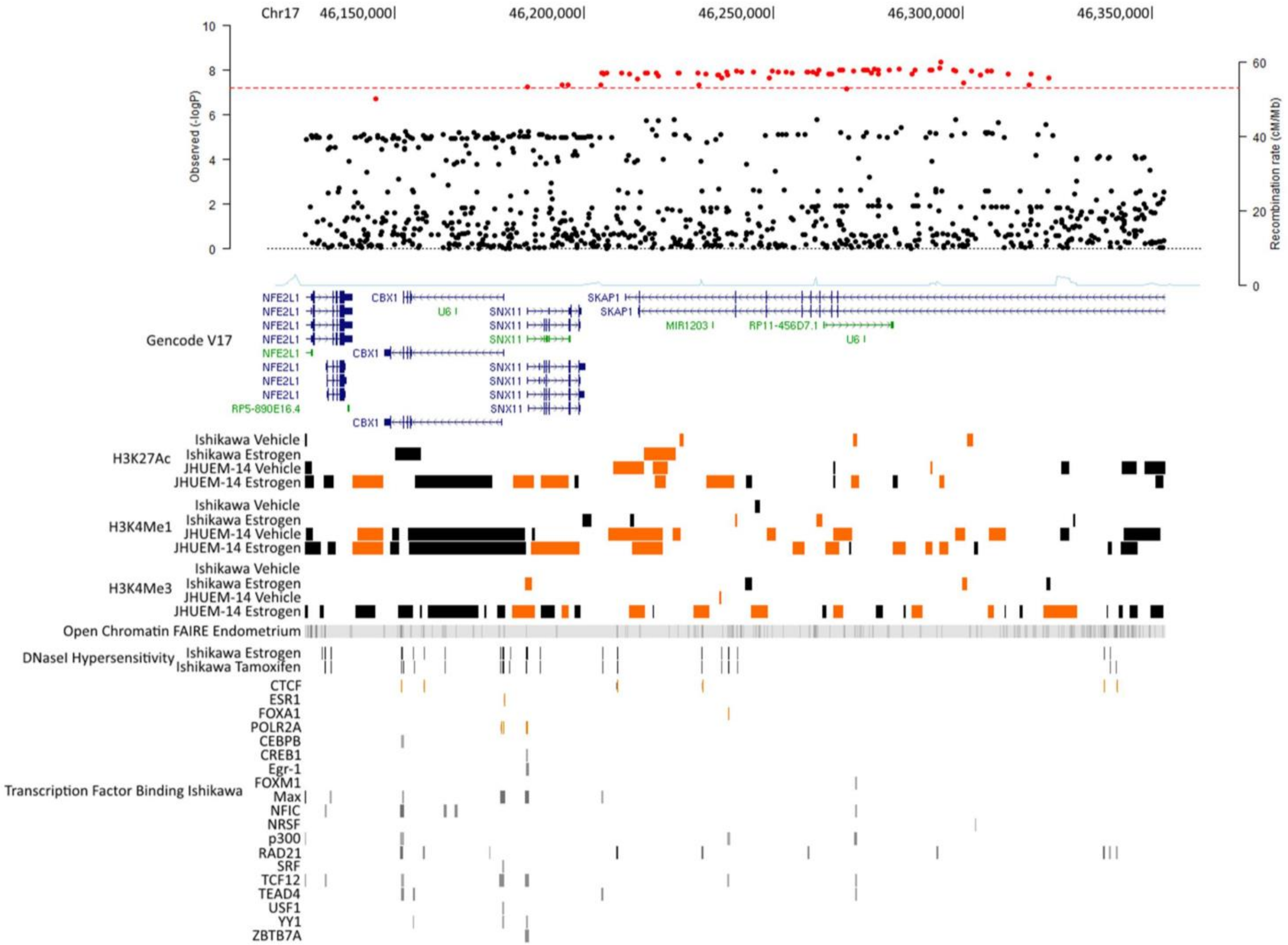

Supplementary Fig. 13. Regional association plots and genomic featured at the 17q21.32 region. ccrSNPs are denoted by red dots. 53 of the 75 ccrSNPs are located in either a DHS or histone mark in endometrial cancer cells (histone marks highlighted in orange). The risk alleles of 30 ccrSNPs are associated with increased *SNX11* expression in blood ($p \leq 8.70 \times 10^{-12}$, ST3) and includes a variant located in the 5'UTR of *SNX11* that is coincident with H3K4Me3 (characteristic of promoters) in both estrogen-treated endometrial cancer cell lines. *SNX11* encodes a vesicular trafficking protein that is involved in nuclear translocation and angiogenesis [17].

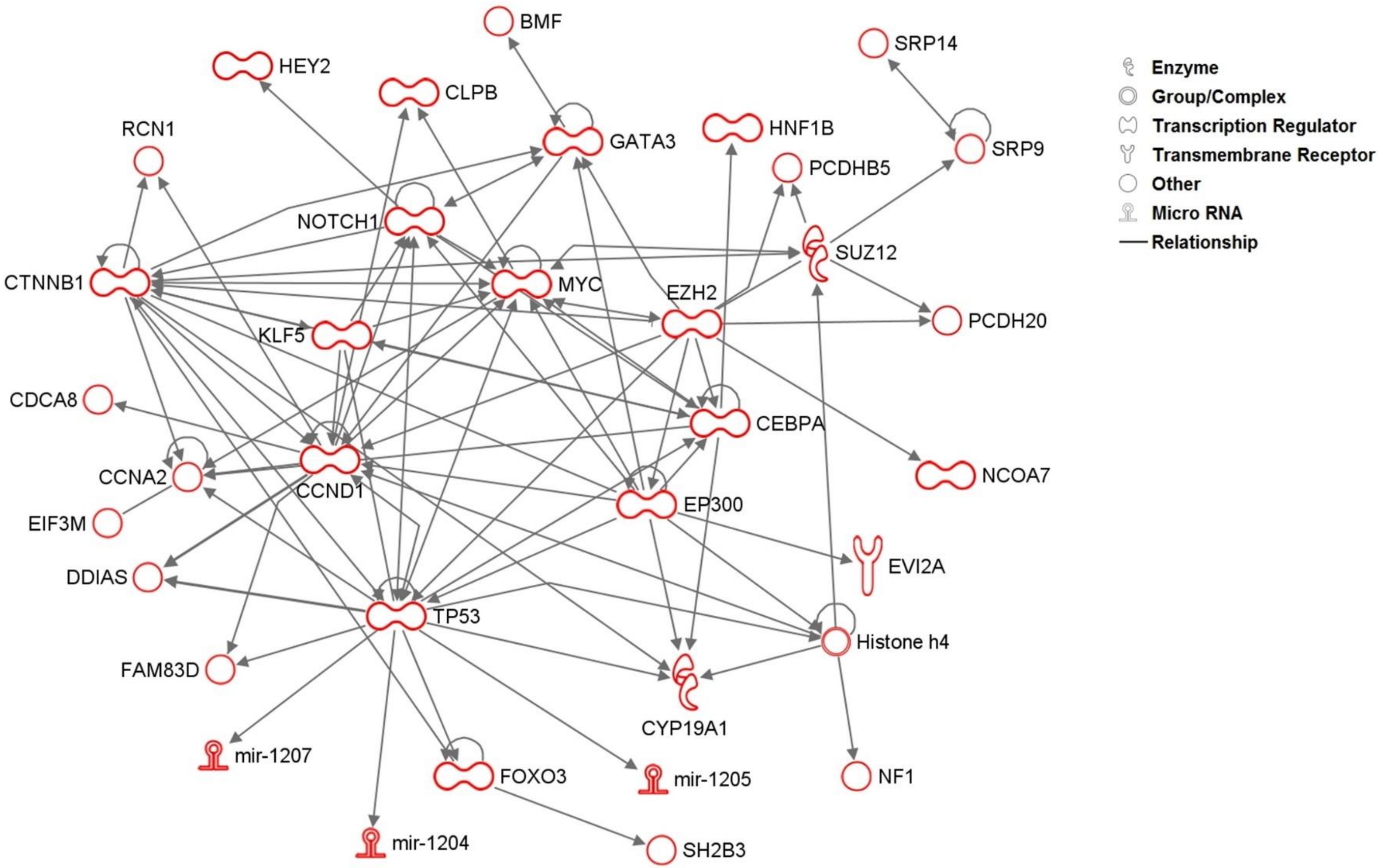

Supplementary Fig. 14. Network analysis of candidate causal genes for endometrial cancer using Ingenuity Pathway Analysis.

Supplementary Table 1. Details for antibodies used for ChIP-seq experiments.

Antibody	Catalogue Number	Concentration	Amount Used
Anti-H3K4Me1	Abcam #ab8895	1 µg/µL	5 µg
Anti-H3K4Me3	Abcam #ab8580	1 µg/µL	5 µg
Anti-H3K27Ac	Abcam #ab4729	1 µg/µL	5 µg

Supplementary Note 1. ECAC Study Collaborators

The ANECS Group comprises: AB Spurdle, PM Webb, J Young (QIMR Berghofer Medical Research Institute); Consumer representative: L McQuire; Clinical Collaborators: NSW: S Baron-Hay, D Bell, A Bonaventura, A Brand, S Braye, J Carter, F Chan, C Dalrymple, A Ferrier (deceased), G Gard, N Hacker, R Hogg, R Houghton, D Marsden, K McIlroy, G Otton, S Pather, A Proietto, G Robertson, J Scurry, R Sharma, G Wain, F Wong; Qld: J Armes, A Crandon, M Cummings, R Land, J Nicklin, L Perrin, A Obermair, B Ward; SA: M Davy, T Dodd, J Miller, M Oehler, S Paramasivum, J Pierides, F Whitehead; Tas: P Blomfield, D Challis; Vic: D Neesham, J Pyman, M Quinn, R Rome, M Weitzer; WA: B Brennan, I Hammond, Y Leung, A McCartney (deceased), C Stewart, J Thompson; Project Managers: S O'Brien, S Moore; Laboratory Manager: K Ferguson; Pathology Support: M Walsh; Admin Support: R Cicero, L Green, J Griffith, L Jackman, B Ranieri; Laboratory Assistants: M O'Brien, P Schultz; Research Nurses: B Alexander, C Baxter, H Croy, A Fitzgerald, E Herron, C Hill, M Jones, J Maidens, A Marshall, K Martin, J Mayhew, E Minehan, D Roffe, H Shirley, H Steane, A Stenlake, A Ward, S Webb, J White.

CHIBCHA (study of hereditary cancer in Europe and Latin America) collaborators include: Ma. Magdalena Echeverry de Polanco, Mabel Elena Bohórquez, Rodrigo Prieto, Angel Criollo, Carolina Ramírez, Ana Patricia Estrada, Jhon Jairo Suárez (Grupo de Citogenética Filogenia y Evolución de Poblaciones, Universidad del Tolima, Colombia); Augusto Rojas Martinez (Center for Research and Development in Health Sciences, Universidad Autónoma de Nuevo León, Monterrey, Mexico); Silvia Rogatto, Samuel Aguiar Jnr, Ericka Maria Monteiro Santos (Department of Urology, School of Medicine, UNESP - São Paulo State University, Botucatu, Brazil); Monica Sans, Valentina Colistro, Pedro C. Hidalgo, Patricia Mut (Department of Biological Anthropology, College of Humanities and Educational Sciences, University of the Republic, Magallanes, Montevideo, Uruguay); Angel Carracedo, Clara Ruiz Ponte, Ines Quntela Garcia (Fundacion Publica Galega de Medicina Xenomica, CIBERER, Genomic Medicine Group-University of Santiago de Compostela, Hospital Clinico, Santiago de Compostela, Galicia, Spain); Sergi Castellvi-Bel (Department of Gastroenterology, Institut de Malalties Digestives i Metabòliques, Hospital Clínic, Centro de Investigación Biomédica en Red de Enfermedades Hepáticas y Digestivas, IDIBAPS, University of Barcelona, Barcelona, Catalonia, Spain); Manuel Teixeira (Department of Genetics, Portuguese Oncology Institute, Rua Dr, António Bernardino de Almeida, Porto, Portugal).

The NSECG Group comprises: Ian Tomlinson; M Adams, A Al-Samarraie, S Anwar, R Athavale, S Awad, A Bali, A Barnes, G Cawdell, S Chan, K Chin, P Cornes, M Crawford, J Cullimore, S Ghaem-Maghami, R Gornall, J Green, M Hall, M Harvey, J Hawe, A Head, J Herod, M Hingorani, M Hocking, C Holland, T Hollingsworth,J Hollingworth, T Ind, R Irvine, C Irwin, M Katesmark, S Kehoe, G Kheng-Chew, K Lankester, A Linder, D Luesley, C B-Lynch,V McFarlane, R Naik, N Nicholas, D Nugent, S Oates, A Oladipo, A Papadopoulos, S Pearson, D Radstone, S Raju, A Rathmell, C Redman, M Rymer, P Sarhanis, G Sparrow, N Stuart, S Sundar, A Thompson, S Tinkler, S Trent, A Tristram, N Walji, R Woolas.

RENDOCAS investigators include: Annika Lindblom, Gerasimos Tzortzatos, Miriam Mints, Emma Tham, Ofra Castro, Kristina Gemzell-Danielsson.

SEARCH collaborators include: Caroline Baynes, Don Conroy, Patricia Harrington, Sue Irvine, Craig Luccarini, Rebecca Mayes.

Supplementary Note 2. BCAC Study Collaborators (for control samples):

ABCFS collaborators: Maggie Angelakos, Judi Maskiell, Gillian Dite

ABCTB collaborators: Rosemary Balleine, Robert Baxter, Stephen Braye, Jane Carpenter, Jane Dahlstrom, John Forbes, Soon Lee, Debbie Marsh, Adrienne Morey, Nirmala Pathmanathan, Allan Spigelman, Nicholas Wilcken, Desmond Yip. Samples are made available to researchers on a non-exclusive basis.

BCEES: Allyson Thomson, Christobel Saunders, Terry Slevin, BreastScreen Western Australia, Elizabeth Wylie, Rachel Lloyd.

BSUCH collaborator: Peter Bugert

ESTHER collaborators: Christa Stegmaier, Katja Butterbach, Katarina Cuk.

GC-HBOC collaborators: Stefanie Engert, Heide Hellebrand, Sandra Kröber and LIFE - Leipzig Research Centre for Civilization Diseases (Markus Loeffler, Joachim Thiery, Matthias Nüchter, Ronny Baber).

GENICA Network collaborators: The GENICA Network: Dr. Margarete Fischer-Bosch-Institute of Clinical Pharmacology, Stuttgart, and University of Tübingen, Germany [HB, Wing-Yee Lo, Christina Justenhoven], German Cancer Consortium (DKTK) and German Cancer Research Center (DKFZ) [HB], Department of Internal Medicine, Evangelische Kliniken Bonn gGmbH, Johanniter Krankenhaus, Bonn, Germany [Yon-Dschun Ko, Christian Baisch], Institute of Pathology, University of Bonn, Germany [Hans-Peter Fischer], Molecular Genetics of Breast Cancer, Deutsches Krebsforschungszentrum (DKFZ), Heidelberg, Germany [Ute Hamann], Institute for Prevention and Occupational Medicine of the German Social Accident Insurance, Institute of the Ruhr University Bochum (IPA), Bochum, Germany [TB, Beate Pesch, Sylvia Rabstein, Anne Lotz]; and Institute of Occupational Medicine and Maritime Medicine, University Medical Center Hamburg-Eppendorf, Germany [Volker Harth].

GESBC collaborator: Ursula Eilber.

HABCS collaborator: Michael Bremer.

LMBC collaborators: Gilian Peuteman, Thomas Van Brussel, EvyVanderheyden and Kathleen Corthouts.

MARIE collaborators: Petra Seibold, Dieter Flesch-Janys, Judith Heinz, Nadia Obi, Alina Vrieling, Sabine Behrens, Ursula Eilber, Muhabbet Celik, Til Olchers and Stefan Nickels.

NBCS collaborators: Dr. Kristine K.Sahlberg, PhD (Department of Research, Vestre Viken Hospital, Drammen, Norway and Department of Cancer Genetics, Institute for Cancer Research, Oslo University Hospital-Radiumhospitalet, Oslo, Norway), Dr. Lars Ottestad, MD (Department of Cancer

Genetics, Institute for Cancer Research, Oslo University Hospital-Radiumhospitalet, Oslo, Norway), Prof. Em. Rolf Kåresen, MD (Institute of Clinical Medicine, University of Oslo, Oslo, Norway and Department of Breast- and Endocrine Surgery, Division of Surgery, Cancer and Transplantation, Oslo University Hospital, Oslo, Norway), Dr. Anita Langerød, PhD (Department of Cancer Genetics, Institute for Cancer Research, Oslo University Hospital-Radiumhospitalet, Oslo, Norway), Dr. Ellen Schlichting, MD (Section for Breast- and Endocrine Surgery, Department of Cancer, Division of Surgery, Cancer and Transplantation Medicine, Oslo University Hospital, Oslo, Norway), Dr. Marit Muri Holmen, MD (Department of Radiology and Nuclear Medicine, Oslo University Hospital, Oslo, Norway), Prof. Toril Sauer, MD (Department of Pathology at Akershus University hospital, Lørenskog, Norway and Institute of Clinical Medicine, Faculty of Medicine, University of Oslo, Oslo, Norway), Dr. Vilde Haakensen, MD (Department of Cancer Genetics, Institute for Cancer Research, Oslo University Hospital-Radiumhospitalet, Oslo, Norway), Dr. Olav Engebråten, MD (Department of Tumor Biology, Institute for Cancer Research, Oslo University Hospital, Oslo, Norway, Department of Oncology, Division of Surgery and Cancer and Transplantation Medicine, Oslo University Hospital, Oslo, Norway and Institute for Clinical Medicine, Faculty of Medicine, University of Oslo, Oslo, Norway), Prof. Bjørn Naume, MD (Department of Oncology, Division of Surgery and Cancer and Transplantation Medicine, Oslo University Hospital-Radiumhospitalet, Oslo, Norway and K.G. Jebsen Centre for Breast Cancer, Institute for Clinical Medicine, University of Oslo, Oslo, Norway.), Dr. Cecile E. Kiserud, MD (National Advisory Unit on Late Effects after Cancer Treatment, Department of Oncology, Oslo University Hospital, Oslo, Norway and Department of Oncology, Oslo University Hospital, Oslo, Norway), Dr. Kristin V. Reinertsen, MD (National Advisory Unit on Late Effects after Cancer Treatment, Department of Oncology, Oslo University Hospital, Oslo, Norway and Department of Oncology, Oslo University Hospital, Oslo, Norway), Assoc. Prof. Åslaug Helland, MD (Department of Genetics, Institute for Cancer Research and Department of Oncology, Oslo University Hospital Radiumhospitalet, Oslo, Norway), Dr. Margit Riis, MD (Dept of Breast- and Endocrine Surgery, Oslo University Hospital, Ullevål, Oslo, Norway), Dr. Ida Bukholm, MD (Department of Breast-Endocrine Surgery, Akershus University Hospital, Oslo, Norway and Department of Oncology, Division of Cancer Medicine, Surgery and Transplantation, Oslo University Hospital, Oslo, Norway), Prof. Per Eystein Lønning, MD (Section of Oncology, Institute of Medicine, University of Bergen and Department of Oncology, Haukeland University Hospital, Bergen, Norway), OSBREAC (Oslo Breast Cancer Research Consortium), Prof. Anne-Lise Børresen-Dale, PhD (Department of Cancer Genetics, Institute for Cancer Research, Oslo University Hospital-Radiumhospitalet, Oslo, Norway and Institute of Clinical Medicine, Faculty of Medicine, University of Oslo, Norway) and Grethe I. Grenaker Alnæs, M.Sc. (Department of Cancer Genetics, Institute for Cancer Research, Oslo University Hospital-Radiumhospitalet, Oslo, Norway).

Supplementary Note 3. Acknowledgements

The authors thank the many individuals who participated in this study and the numerous institutions and their staff who have supported recruitment.

ANECS thanks members of the Molecular Cancer Epidemiology and Cancer Genetic laboratories at QIMR Berghofer Medical Research Institute for technical assistance, and the ANECS research team for assistance with the collection of risk factor information and blood samples. ANECS also gratefully acknowledges the cooperation of the following institutions: NSW: John Hunter Hospital, Liverpool Hospital, Mater Misericordiae Hospital (Sydney), Mater Misericordiae Hospital (Newcastle), Newcastle Private Hospital, North Shore Private Hospital, Royal Hospital for Women, Royal Prince Alfred Hospital, Royal North Shore Hospital, Royal Prince Alfred Hospital, St George Hospital; Westmead Hospital, Westmead Private Hospital; Qld: Brisbane Private Hospital, Greenslopes Hospital, Mater Misericordiae Hospitals, Royal Brisbane and Women's Hospital, Wesley Hospital, Queensland Cancer Registry; SA: Adelaide Pathology Partners, Burnside Hospital, Calvary Hospital,

Flinders Medical Centre, Queen Elizabeth Hospital, Royal Adelaide Hospital, South Australian Cancer Registry; Tas: Launceston Hospital, North West Regional Hospitals, Royal Hobart Hospital; Vic: Freemasons Hospital, Melbourne Pathology Services, Mercy Hospital for Women, Royal Women's Hospital, Victorian Cancer Registry; WA: King Edward Memorial Hospital, St John of God Hospitals Subiaco & Murdoch, Western Australian Cancer Registry.
SEARCH thanks the SEARCH research team for recruitment, and also acknowledges the assistance of the Eastern Cancer Registration and Information Centre for subject recruitment.
BECS thanks Reiner Strick, Silke Landrith and Sonja Oeser for their logistic support during the study.
CAHRES (formerly known as SASBAC) thanks Li Yuqing from the Genome Institute of Singapore for contributions to this study, and also acknowledges previous input to SASBAC resource creation by Anna Christensson, Boel Bissmarck, Kirsimari Aaltonen, Karl von Smitten, Nina Puolakka, Christer Halldén, Lim Siew Lan and Irene Chen, Lena U. Rosenberg, Mattias Hammarström, and Eija Flygare.
HJECS thanks Wen Zheng, Hermann Hertel, and Tjoung-Won Park-Simon at Hannover Medical School for their contribution to sample recruitment.
LES gratefully acknowledges Helena Soenen, Gilian Peuteman and Dominiek Smeets for their technical assistance.
MCCS was made possible by the contribution of many people, including the original investigators and the diligent team who recruited the participants and who continue working on follow up. We would like to express our gratitude to the many thousands of Melbourne residents who continue to participate in the study.
MECS thanks Tom Sellers, Catherine Phelan, Andrew Berchuck, and Kimberly Kalli, Amanda von Bismarck, Luisa Freyer and Lisa Rogmann.
MoMaTEC thanks Britt Edvardsen, Ingjerd Bergo and Mari Kyllsø Halle for technical assistance and Inger Marie Aksnes and Tor Audun Hervig at the Blood bank, Haukeland University Hospital for assistance with control recruitment.
NECS thanks staff at the University of Newcastle and the Hunter Medical Research Institute.
NSECG thank Ella Barclay and Lynn Martin for their contribution, and acknowledge the invaluable help of the National Cancer Research Network with the collection of study participants.
RENDOCAS thanks Berith Wejderot, Sigrid Sahlen, Tao Liu, Margareta Ström, Maria Karlsson, and Birgitta Byström for their contribution to the study.
BSUCH thanks the Medical Faculty, Mannheim, the Diemtmar Hopp Foundation and the German Cancer Research Center.
MMHS thank the coordinators, the research staff and especially the participants for their continued collaboration on research studies in breast cancer.
WHI thank the investigators and staff for their dedication and the study participants for making the program possible.
UKBGS thank Breakthrough Breast Cancer and the Institute of Cancer Research for support and funding, and the Study participants, Study staff, and the doctors, nurses and other health care staff and data providers who have contributed to the Study. The ICR acknowledges NHS funding to the NIHR Biomedical Research Centre.

In addition, the iCOGS study would not have been possible without the contributions of: Andrew Berchuck (OCAC), Rosalind A. Eeles, Ali Amin Al Olama, Zsofia Kote-Jarai , Sara Benlloch (PRACTICAL), Georgia Chenevix-Trench, Antonis Antoniou, Lesley McGuffog, Ken Offit (CIMBA), Andrew Lee, and Ed Dicks, Craig Luccarini and the staff of the Centre for Cancer Genetic Epidemiology Laboratory (Cambridge), Javier Benitez, Anna Gonzalez-Neira and the staff of the CNIO genotyping unit, Jacques Simard and Daniel C. Tessier, Francois Bacot, Daniel Vincent, Sylvie LaBoissière and Frederic Robidoux and the staff of the McGill University and Génome Québec Innovation Centre, Stig E. Bojesen, Sune F. Nielsen, Borge G. Nordestgaard, and the staff of the Copenhagen DNA laboratory, Sharon A. Windebank, Christopher A. Hilker, Jeffrey Meyer and the staff of Mayo Clinic Genotyping Core Facility.

Supplementary Note 4. Acknowledgments of Funding to BCAC control groups

The Australian Breast Cancer Family Study (ABCFS) was supported by grant UM1 CA164920 from the National Cancer Institute (USA). The content of this manuscript does not necessarily reflect the views or policies of the National Cancer Institute or any of the collaborating centers in the Breast Cancer Family Registry (BCFR), nor does mention of trade names, commercial products, or organizations imply endorsement by the USA Government or the BCFR. The ABCFS was also supported by the National Health and Medical Research Council of Australia, the New South Wales Cancer Council, the Victorian Health Promotion Foundation (Australia) and the Victorian Breast Cancer Research Consortium. J.L.H. is a National Health and Medical Research Council (NHMRC) Senior Principal Research Fellow. M.C.S. is a NHMRC Senior Research Fellow.

The Australian Breast Cancer Tissue Bank (ABCTB) is generously supported by the National Health and Medical Research Council of Australia, The Cancer Institute NSW and the National Breast Cancer Foundation.

The work of the BBCC was partly funded by ELAN-Fond of the University Hospital of Erlangen.

The BCEES was funded by the National Health and Medical Research Council, Australia and the Cancer Council Western Australia and acknowledges funding from the National Breast Cancer Foundation (JS).

The ESTHER study was supported by a grant from the Baden Württemberg Ministry of Science, Research and Arts.

The GC-HBOC (German Consortium of Hereditary Breast and Ovarian Cancer) is supported by the German Cancer Aid (grant no 110837, coordinator: Rita K. Schmutzler, Cologne). This work was also funded by the European Regional Development Fund and Free State of Saxony, Germany (LIFE - Leipzig Research Centre for Civilization Diseases, project numbers 713-241202, 713-241202, 14505/2470, 14575/2470).

The GENICA was funded by the Federal Ministry of Education and Research (BMBF) Germany grants 01KW9975/5, 01KW9976/8, 01KW9977/0 and 01KW0114, the Robert Bosch Foundation, Stuttgart, Deutsches Krebsforschungszentrum (DKFZ), Heidelberg, the Institute for Prevention and Occupational Medicine of the German Social Accident Insurance, Institute of the Ruhr University Bochum (IPA), Bochum, as well as the Department of Internal Medicine, Evangelische Kliniken Bonn gGmbH, Johanniter Krankenhaus, Bonn, Germany.

The GESBC was supported by the Deutsche Krebshilfe e. V. [70492] and the German Cancer Research Center (DKFZ).

The HABCS study was supported by by the Claudia von Schilling Foundation for Breast Cancer Research, by the Lower Saxonian Cancer Society, and by the Rudolf Bartling Foundation.

The KARMA study was supported by Märit and Hans Rausings Initiative Against Breast Cancer.

LMBC is supported by the 'Stichting tegen Kanker'. Diether Lambrechts is supported by the FWO.

The MARIE study was supported by the Deutsche Krebshilfe e.V. [70-2892-BR I, 106332, 108253, 108419, 110826, 110828], the Hamburg Cancer Society, the German Cancer Research Center (DKFZ)

and the Federal Ministry of Education and Research (BMBF) Germany [01KH0402].

The MCBCS was supported by the NIH grants CA192393, CA116167, CA176785 an NIH Specialized Program of Research Excellence (SPORE) in Breast Cancer [CA116201], and the Breast Cancer Research Foundation and a generous gift from the David F. and Margaret T. Grohne Family Foundation.

MCCS cohort recruitment was funded by VicHealth and Cancer Council Victoria. The MCCS was further supported by Australian National Health and Medical Research Council grants 209057 and 396414 and by infrastructure provided by Cancer Council Victoria. Cases and their vital status were ascertained through the Victorian Cancer Registry and the Australian Institute of Health and Welfare, including the National Death Index and the Australian Cancer Database.

The MISS study is supported by funding from ERC-2011-294576 Advanced grant, Swedish Cancer Society, Swedish Research Council, Local hospital funds, Berta Kamprad Foundation, Gunnar Nilsson.

The NBCS has been supported by the Research Council of Norway grant 193387/V50 (to A-L Børresen-Dale and V.N. Kristensen) and grant 193387/H10 (to A-L Børresen-Dale and V.N. Kristensen), South Eastern Norway Health Authority (grant 39346 to A-L Børresen-Dale and 27208 to V.N.Kristensen) and the Norwegian Cancer Society (to A-L Børresen-Dale and 419616 - 71248 - PR-2006-0282 to V.N. Kristensen). It has received funding from the K.G. Jebsen Centre for Breast Cancer Research (2012-2015).

The SMC is funded by the Swedish Cancer Foundation.

UKBGS was funded by Breakthrough Breast Cancer and the Institute of Cancer Research, which acknowledges NHS funding to the NIHR Biomedical Research Centre

Supplementary References

1. Hayama, S. *et al.* Phosphorylation and activation of cell division cycle associated 8 by aurora kinase B plays a significant role in human lung carcinogenesis. *Cancer Res* **67**, 4113-4122 (2007).
2. Wang, Y. *et al.* Borealin/Dasra B is overexpressed in colorectal cancers and contributes to proliferation of cancer cells. *Med Oncol* **31**, 248 (2014).
3. Wrzeszczynski, K.O. *et al.* Identification of Tumor Suppressors and Oncogenes from Genomic and Epigenetic Features in Ovarian Cancer. *PLOS ONE* **6**, e28503 (2011).
4. Lawrenson, K. *et al.* Cis-eQTL analysis and functional validation of candidate susceptibility genes for high-grade serous ovarian cancer. *Nat Commun* **6**, 8234 (2015).
5. May, E.W. *et al.* Identification of up- and down-regulated proteins in doxorubicin-resistant uterine cancer cells: reticulocalbin-1 plays a key role in the development of doxorubicin-associated resistance. *Pharmacol Res* **90**, 1-17 (2014).
6. Ren, Y., Yeoh, K.W., Hao, P., Kon, O.L. & Sze, S.K. Irradiation of Epithelial Carcinoma Cells Upregulates Calcium-Binding Proteins That Promote Survival under Hypoxic Conditions. *Journal of Proteome Research* **15**, 4258-4264 (2016).
7. McCarty, G. & Loeb, D.M. Hypoxia-Sensitive Epigenetic Regulation of an Antisense-Oriented lncRNA Controls WT1 Expression in Myeloid Leukemia Cells. *PLOS ONE* **10**, e0119837 (2015).
8. Moorwood, K. *et al.* Antisense WT1 transcription parallels sense mRNA and protein expression in fetal kidney and can elevate protein levels in vitro. *J Pathol* **185**, 352-359 (1998).
9. Miller-Hodges, E. & Hohenstein, P. WT1 in disease: shifting the epithelial-mesenchymal

balance. *J Pathol* **226**, 229-240 (2012).

10. Wu, W. *et al.* Tissue-specific Co-expression of Long Non-coding and Coding RNAs Associated with Breast Cancer. **6**, 32731 (2016).
11. Devalliere, J. & Charreau, B. The adaptor Lnk (SH2B3): an emerging regulator in vascular cells and a link between immune and inflammatory signaling. *Biochem Pharmacol* **82**, 1391-1402 (2011).
12. Gevaert, O., Villalobos, V., Sikic, B.I. & Plevritis, S.K. Identification of ovarian cancer driver genes by using module network integration of multi-omics data. *Interface Focus* **3**(2013).
13. Simanshu, D.K., Nissley, D.V. & McCormick, F. RAS Proteins and Their Regulators in Human Disease. *Cell* **170**, 17-33 (2017).
14. Margueron, R. & Reinberg, D. The Polycomb complex PRC2 and its mark in life. *Nature* **469**, 343-349 (2011).
15. Chiang, S. *et al.* Frequency of known gene rearrangements in endometrial stromal tumors. *Am J Surg Pathol* **35**, 1364-1372 (2011).
16. Ma, X. *et al.* The JAZF1-SUZ12 fusion protein disrupts PRC2 complexes and impairs chromatin repression during human endometrial stromal tumorogenesis. *Oncotarget* **8**, 4062-4078 (2017).
17. Joyal, J.S. *et al.* Subcellular localization of coagulation factor II receptor-like 1 in neurons governs angiogenesis. *Nat Med* **20**, 1165-1173 (2014).
