# Supplementary material for: Identification of nine new susceptibility loci for endometrial cancer
Source: Nat Commun. 2018 Aug 9;9:3166. doi: 10.1038/s41467-018-05427-7 (PMC6085317; doi:10.1038/s41467-018-05427-7)
Supplement: Supplementary file 3 — Description of Additional Supplementary Files [file 41467_2018_5427_MOESM3_ESM.pdf]

Description of Additional Supplementary Files

File Name: Supplementary Data 1
Description: Details of sample sets contributing to the endometrial cancer genetic association meta-analysis.

File Name: Supplementary Data 2
Description: Lead SNPs for all loci for which at least one SNP has meta-analysis $P<1x10^{-6}$ for the all histologies analysis, for the endometrioid-only analysis or for the non-endometrioid histologies analysis.

File Name: Supplementary Data 3
Description: Candidate Causal Risk SNPs (ccrSNPs) for the novel risk loci and intersections with functional genomic features.

File Name: Supplementary Data 4
Description: Published genome-wide significant associations for cancer and hormonal/reproductive/anthropometric phenotypes within 500kb of the top hit at the newly identified endometrial cancer loci. Data from NHGRI-EBI catalog of published genome-wide association studies (https://www.ebi.ac.uk/gwas/) accessed on 11-05-2017.

File Name: Supplementary Data 5
Description: Expression quantitative trait loci (eQTL) summary statistics for Candidate Causal Risk SNPs (ccrSNPs) identified as eQTLs from blood, endometrium or endometrial cancer tumour tissue.

File Name: Supplementary Data 6
Description: Candidate causal genes for network analysis

File Name: Supplementary Data 7
Description: Biological pathway enrichment analysis of candidate causal gene network

File Name: Supplementary Data 8
Description: Genetic correlation between endometrial cancer and 224 traits from LDHub
